# Dynamics of agricultural land and the risk to food insecurity in the *Niayes* region of Diamniadio, West Senegal

**DOI:** 10.4102/jamba.v9i1.355

**Published:** 2017-07-26

**Authors:** Mateugue Diack, Macoumba Loum, Abdoulaye Guisse, Mamadou B. Sane

**Affiliations:** 1 Faculty of Agricultural Sciences, Aquaculture and Food Technology, Gaston Berger University, Senegal; 2 Department of Political and Legal Sciences, Gaston Berger University, Senegal; 3 Faculty of Letters and Human Sciences, Gaston Berger University, Senegal

## Abstract

Food security is a serious challenge facing West African countries because most croplands are being degraded. Consequently, agricultural production is being exceeded by rapid population growth. This study relates the dynamics of agricultural lands to the level of capacity building for resilience in response to low productivity and hence to food insecurity in the *Niayes* region, Senegal, where lands are presumably suitable for crop production. Factors influencing changes in surface areas, soil quality and level of resilience were examined using quantitative and qualitative research methods. Findings showed strong relationships between a significant decrease (65.25% – 35.54%) in productive agricultural lands with a range of soil physical and chemical properties (clay to loamy soil texture; soil pH: 7.0–8.0; soil organic carbon [SOC]: 5.0 g kg^−1^ – 25.0 g kg^−1^; effective cations exchangeable capacity [ECEC]: 4.5 Cmol kg^−1^ – 39.0 Cmol kg^−1^; cation exchange capacity [CEC]: 8.0 Cmol kg^−1^ – 34.0 Cmol kg^−1^) and food insecurity levels. In the last 5 years, urbanisation and industrialisation processes have reduced the farmlands by about 26.51% through uncontrolled construction of buildings and companies, leading to a disappearance of lands. Such dynamics raises the issue of a risk to food security in a region that usually provides more than 70% of fruits and vegetables demand for consumption. These results underline a need for a greater understanding of resilience for a better management design with a risk prevention plan to ensure food security.

## Introduction

**FIGURE 1:**
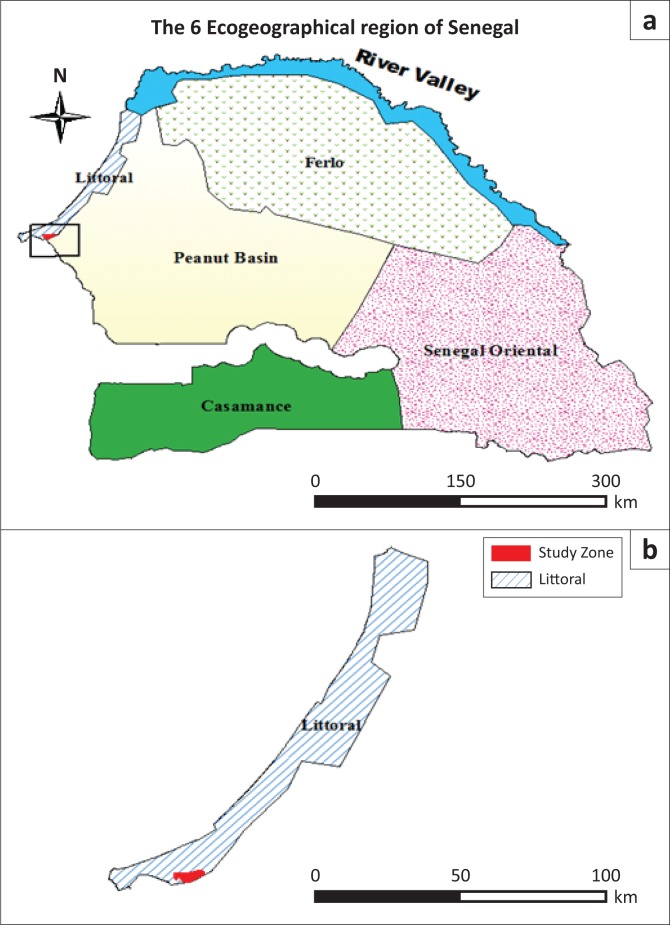
Location of the study area. (a) The Niayes region within Senegal and (b) within the littoral.

In the semi-arid regions of Africa, agriculture provides food, income, power, stability and resilience to rural livelihoods (Challinor et al. 2006). In Senegal, public policies and private initiatives were carried out after independence for sustaining farmers in order to achieve food security. During the 1970s, the company of BUD-Senegal and the *Centre pour le Développement de l’Horticulture* initiated the intensification of the horticulture sector in the *Niayes* region (Wade, Benz & Egg 2004). The *Niayes* region is the eco-geographical area of the Senegalese coast, of about 180 km long and 25 km wide. It is bounded to the west by the Atlantic Ocean and to the east by the Dakar/Saint-Louis national highway (Figure 1). The landscape components include depressions and dunes with shape process going back to the periods of quaternary and tertiary. The dominant soil types are sandy soils, vertisols and hydromorphic soils (Ndao 2012). Moisture conditions allowed the accumulation of soil organic matter in the depressions. Natural vegetation is characterised by wetland species (*Eleais guineensis, Prosopis africana*) and savanna species (*Adansonia digitata, Acacia* spp.; Faye 2010). Market gardening, arboriculture and fishing are the main socio-economic activities. Until 2004, 60% of the national needs for consumption of vegetables were produced from the *Niayes* region, as well as 80% of the exports of horticultural products (Seck et al. 2005). The richness of the biodiversity and the proximity to the sea give the *Niayes* area a microclimate with more moderate temperatures compared to the climate in the regions located in the mainland. Such biophysical conditions have created a favourable living environment in the *Niayes* region and therefore have led to ineluctable change in a diverse range of land uses. Consequently, agricultural lands tend to decrease in favour of the construction of buildings and industrial units. This situation was a serious concern because the *Niayes* region, intended primarily for market gardening activities, is the smallest of the six eco-geographical zones of Senegal. It represents about 2% of the surface area of the country. These spatial dynamics tend to affect the performance of horticultural activities. In the Sahel, the risk of food insecurity related to climate conditions was emphasised (Benson & Clay 1998; Cooper et al. 2008; Giannini, Biasutti & Verstraete 2008; Leroux 1995; Toupet 1995). Such a situation resulted from the drought of the 1970s that revealed the vulnerability of agricultural production systems in the Sahel (FAO 2012). However, human activities are often factors of imbalance in the agro-system organisation. Because of favourable climatic conditions for human development, construction of buildings tends more and more to monopolise land use in the *Niayes* region. Industrial activities are also expanding. This urbanisation and industrialisation process causes the loss of agricultural land and constitutes a threat to food security because of a high contribution of market gardening and horticultural products in food demand in Senegal. Certainly, rice and millet are the basic cereals for local consumption. Nevertheless, onion, carrot, cabbage, eggplant, tomato, pepper, beans, okra and cassava, also produced in the *Niayes* region, are various condiments widely consumed by people in Senegal. Watermelon, melon, mango and citrus fruits, which come from the rural *Niayes* areas, are also consumed nationally. In this regard, the *Niayes* region is the field of predilection for vegetable and fruits production, therefore justifying the analysis of the risk in relation to the dynamic of land use. The risk analysis uses the concepts of hazard, vulnerability and challenge (Brooks 2003; Cummins & Mahul 2008; Veyret & Reghezza 2005). In this study, urbanisation and industrialisation represent the hazard. Agricultural areas reflect the vulnerability and their dynamics lead to food insecurity (Brooks 2003; World Bank 2010b). Furthermore, the construction of an urban pole in Diamniadio by public authorities, with the implementation of major infrastructures like the *Centre International de Conferences Abdou Diouf* (*CICAD*), the *Université Amadou Moctar MBow* of Diamniadio or the turnpike road, has markedly increased the loss of arable land. Thus, through a linear and prospective analysis of the dynamics of agricultural lands of the study area and its potential for agricultural productivity, it is intended to provide indicators of the level of risk for food insecurity. The objective of this study is to determine the level of risk of food insecurity with regards to the dynamics of land use and soil fertility within the *Niayes* region.

## Materials and method

### The study area

**FIGURE 2:**
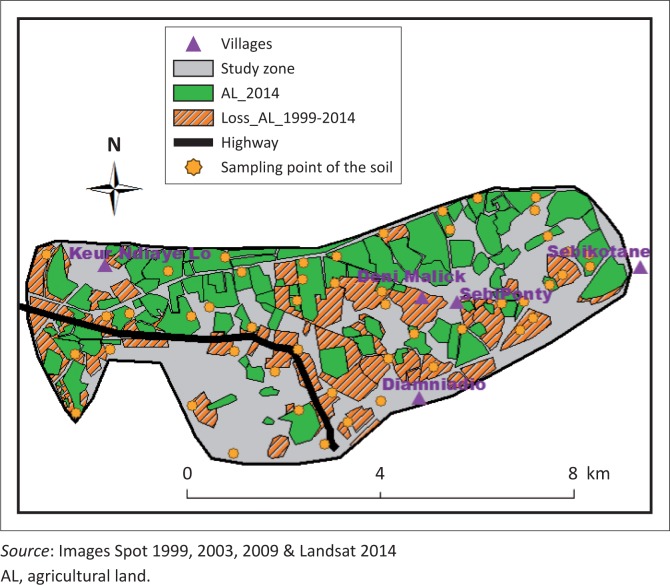
Dynamics of the agricultural land from 1999 to 2014.

The study area corresponds to 4360 ha of land including the villages of Sébikotane (SBK), SébiPonty (SBP), Deni Malick Gueye (DMG) and Keur Ndiaye Lo (KNL) (Figure 2). The climate presents oceanic features. The maritime trade winds and ocean currents mitigate seasonal thermal contrasts of the sahelian climate (Aguiar 2009). The rainy season lasts 4 months (July–October). The temperatures were recorded with a maximum of 32 °C (Table 1). During dry seasons with relatively low temperatures (December through February), the minimum can decrease to 17 °C (Table 2). The relative humidity ranged from 25% in December and January to 95% in March and April (ANACIM 2015).

**TABLE 1:** Monthly means of maximum temperatures (°C).

Year	Month											
	Jan.	Feb.	Mar.	Apr.	May	June	July	Aug.	Sept.	Oct.	Nov.	Dec.
2009	24.1	22.6	23.4	24.5	26.2	29.2	30.9	31.0	30.9	31.6	30.4	27.7
2010	27.5	27.0	27.5	26.6	27.4	29.9	31.2	31.3	31.1	31.3	31.4	29.0
2011	27.9	26.0	23.9	25.4	25.7	29.1	30.4	30.8	31.4	32.3	29.4	28.6
2012	26.2	23.4	26.0	24.1	26.5	28.6	29.7	30.2	30.7	31.9	30.4	26.6
2013	25.9	26.8	25.5	30.4	27.7	28.9	30.4	30.4	30.4	30.4	28.8	26.5
2014	24.4	30.4	23.5	24.6	25.3	29.2	30.4	30.7	31.0	31.5	29.2	26.9

*Source:* National Agency for Civil Aviation and Meteorology (ANACIM 2015)

**TABLE 2:** Monthly means of minimum temperatures (°C).

Year	Month											
	Jan.	Feb.	Mar.	Apr.	May	June	July	Aug.	Sept.	Oct.	Nov.	Dec.
2009	17.7	17.0	17.8	20.9	20.2	23.5	25.4	24.7	25.0	26.1	23.6	21.3
2010	18.7	19.0	20.0	20.9	21.7	26.6	25.9	25.8	25.1	25.7	24.0	23.3
2011	21.2	17.8	17.9	19.4	20.5	23.9	25.3	25.5	25.5	26.2	23.8	21.9
2012	19.0	19.7	18.7	18.9	21.1	23.8	25.4	25.0	25.3	25.9	24.9	20.6
2013	21.7	18.8	19.0	19.1	21.6	24.0	25.7	25.5	25.3	26.2	23.3	20.7
2014	18.4	16.5	17.7	18.9	20.7	24.2	25.9	25.9	25.6	26.5	24.6	21.2

*Source:* National Agency for Civil Aviation and Meteorology (ANACIM 2015)

### Processing of satellite data

The quantification of agricultural land dynamics is based on remote sensing data. A three-satellite image of SPOT composed of one image in 1999, a second image in 2003 and a third image in 2009 was used. The processing of the Landsat image used in 2014 has required ground control point collected with GPS in the field, whereas the images of SPOT are sufficiently accurate for the monitoring of the land use dynamic (resolution between 2.5 m and 10.0 m). Remote sensing data have given satisfactory results related to the linear analysis of the evolution of Sahel areas (Loum 2012; Ndao 2012; Poncet 1986). The processing of satellite images (SPOT and LANDSAT), with Envi and ArcGIS software associated with GPS collections for training data, has allowed the monitoring of the dynamics of agricultural lands in the study area. A linear prediction model of the future dynamics of agricultural areas in the *Niayes* is also applied to better highlight the risk to food security.

### Soil sampling and analysis

The description of the soil pits coupled with the soil sample collected by auger was used to analyse the physical and chemical properties of the soil. Sampling points are identified by GPS and visualised through a Geographic Information System. The dimensions of the soil pits are 1.5 m long, 1.0 m wide and 2.0 m deep. These pits are dug in residential areas as well as in the agricultural land plots. Training points are also positioned in consideration of this type of land distribution. Soil samples were taken at the counsel of 0 cm – 10 cm, 10 cm – 20 cm, 20 cm – 40 cm, 40 cm – 60 cm and 60 cm – 80 cm depth. Soil samples were analysed for soil acidity (measuring soil pH), soil organic carbon (SOC), effective cations exchangeable capacity (ECEC), as the sum of calcium, magnesium, potassium and sodium, and cation exchange capacity (CEC). Soil pH was determined in duplicate in distilled water using a soil/liquid ratio of 1:2.5. After stirring for 30 min, the pH value was read off using a glass electrode pH meter (Mclean 1982). SOC was obtained by the wet dichromate acid oxidation method (Nelson & Sommers 1982). Exchangeable basic cations were extracted using 1N NH_4_OAC at pH 7. Calcium concentration was measured by atomic absorption spectrophotometry. Particle size distribution was determined using the hydrometer method (Gee & Bauder 1986).

## Results

### The satellite data

The training data collected with GPS have firstly allowed digitising and interpreting changes in land use with the Landsat image of 2014. Thus, in 2014, the area of the agricultural land was 1541 ha (Figure 2). Secondly, the retrospective dynamics of agricultural land was reconstructed through a finer resolution of SPOT images (10 m for SPOT 4, 5 m for SPOT 5 m and 2.5 m for SPOT 6).

### Dynamics of the agricultural land

**FIGURE 3:**
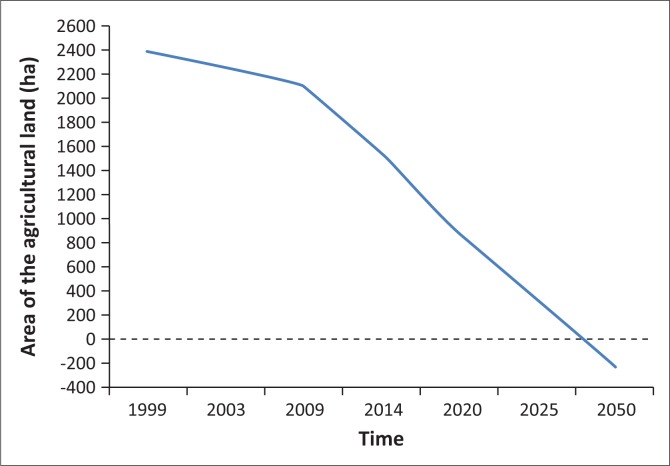
Prospective changes in agricultural lands (1999–2050).

**FIGURE 4:**
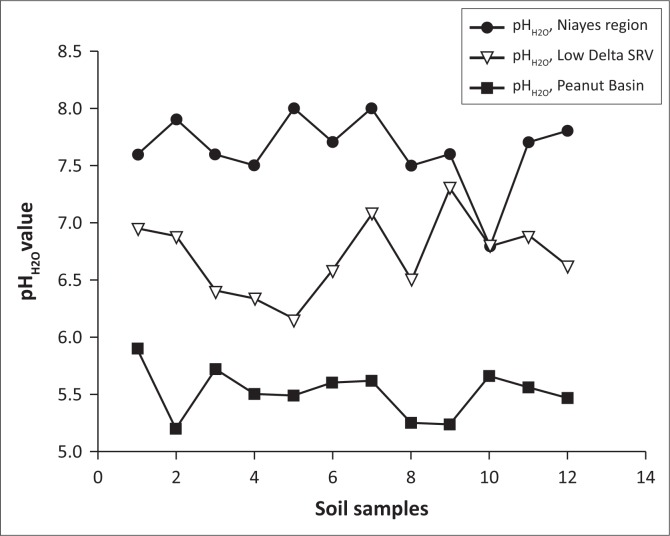
Comparison of the soil pH_H2O_ values in the *Niayes* region, the Peanut Basin and the Senegal River Valley.

The results showed 2384 ha in 1999, then 2256 ha in 2003, 2097 ha in 2009 and finally 1541 ha in 2014. For a study area of 4360 ha, the agricultural land varied from 54.68% in 1999 to 35.34% in 2014 (Figure 3). On average, the area of the agricultural land decreased at a rate of 110 ha per year between 2009 and 2014. Assuming a linear evolution of the dynamics of the land use, the results highlighted a risk of disappearance of agricultural lands in the *Niayes* region of Diamniadio towards 2050. In 2025, the area of cropland remaining is predicted to be 300 ha (Figure 4).

### Soil quality assessment

Dynamics of the agricultural lands need to be related to the soil quality assessment in order to better understand the level of risk related to food security. Across the four villages, the description of soil profiles from the pits shows different layers with soil depths varying from 36 cm to 124 cm and a mean depth of 79.5 cm (Table 3). From the observed layers, soil profiles have for the most part A, AB or BC horizons, showing a greater concentration of clay than sand in the agricultural lands. Colour of the soils, from the surface down to the deep horizons, varies from 2.5YR (light reddish brown) to 7YR (greyish brown) and to 10YR (dark greyish brown). The texture ranges from loamy to clayey soil in the soil profile. The soil structure varies from massive and angular to polyhydric status within the soil profile and throughout the four villages.

**TABLE 3:** Description of the soil profiles at SébiPonty, Sébikotane, Deni Malick and Keur Ndiaye Lo villages.

Pits	Depth	Horizon	Colour	Texture	Structure
SBP1	0 cm – 77 cm: presence of groundwater	A	10YR 4/1; 10YR 2/2	Silty clay	Angular
SBP2	0 cm – 43 cm	AB1	10YR 4/1; 10YR3/1	Silty clay	Angular
	43 cm – 70 cm	AB2	10YR 6/6	Silty clay	Angular
	+70 cm	BC	2.5YR 5/4	Silty clay	Angular
SBK1	0 cm – 67 cm	AB1	10YR 4/1	Silty clay	Polyhydric
	67 cm – 96 cm	AB2	10YR 4/1; 10YR 2/1	Silty clay	Polyhydric to angular
	96 cm +	BC	2.5YR 8/4	Silty clay	Angular
SBK2	0 cm – 60 cm	AB1	10YR 3/1; 10YR 5/1	Silty clay	Polyhydric
	60 cm – 115 cm	AB2	7.5YR 2/0; 7.5YR 2/0	Silty clay	Polyhydric
	+115 cm	BC	2.5YR 8/2; 2.5YR 8/3	Silty clay	Angular
DM1	0 cm – 37 cm	AB1	10YR 3/2	Silty clay	Angular
	37 cm – 60 cm	AB2	2.5YR6/6	Silty clay	Angular
	+60 cm	BC	2.5Y8/2; 2.5Y7/6	Clayey	Polyhydric
DM2	0 cm – 36 cm	AB1	10YR3/2	Sandy clay	Angular to massive
	+36 cm	AB2	10YR 4/2	Silty clay	Angular
KNL1	0 cm – 30 cm	AB1	10YR 3/1	Silty clay	Polyhydric to angular
	30 cm – 41 cm	AB2	10YR 4/2	Silty clay	Angular
	41 cm – 60 cm	BC	10YR 3/2	Sandy clay	Angular to massive
	60 cm – 124 cm	C1	10YR 8/2	Sandy clay	Particular
	+124 cm	BC	10YR 7/8	Sandy clay	Angular to massive
KNL2	0 cm – 10 cm	AB1	7.5YR 6/6	Sandy clay	Angular to massive
	10 cm – 58 cm	AB2	7.5YR 5/1	Silty clay	Polyhydric
	+58 cm	AB3	10YR 4/1	Silty clay	Polyhydric

SBP, SébiPonty; SBK, Sébikotane; DM, Deni Malick; KNL, Keur Ndiaye Lo.

The pH values of the soil ranged between 7 and 8. Salinity is less important with sodium contents of 0.5 Cmol kg^−1^ of soil. The range of organic carbon content was between 5 g kg^−1^ and 25 g kg^−1^.

Soil texture is predominately of clay then loamy with fine fraction content between 60% and 90%. Sandy soils represent about 10% of the texture. Calcium contents ranged from 4 Cmol kg^−1^ to 25 Cmol kg^−1^ of soil. The variability of the magnesium was between 0.5 Cmol kg^−1^ and 14 Cmol kg^−1^ of soil. The CEC, whose values were between 8 Cmol kg^−1^ and 34 Cmol kg^−1^, showed appreciated characteristics of the clay-humic complex.

## Discussion

**FIGURE 5:**
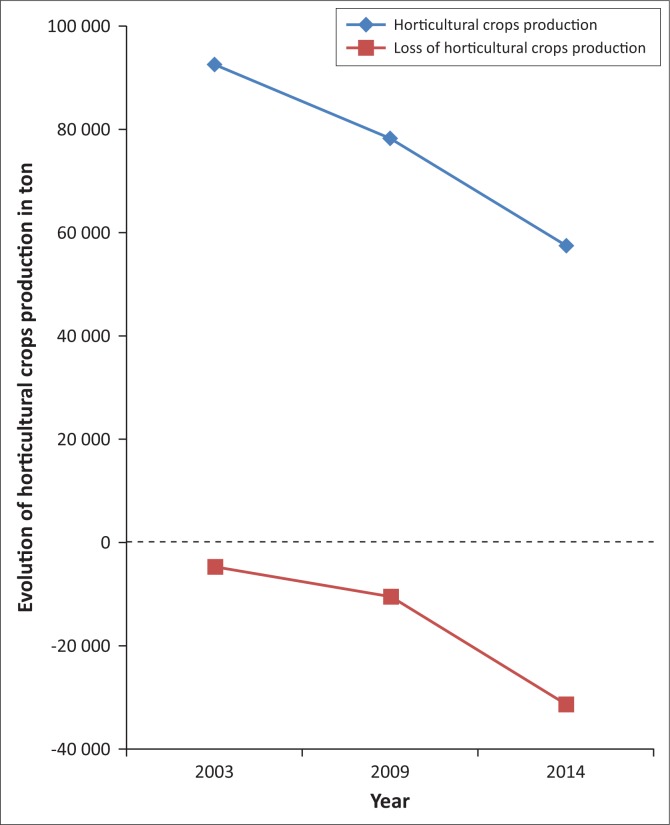
Changes in horticultural crops production as affected by the urbanisation and industrialisation process.

**FIGURE 6:**
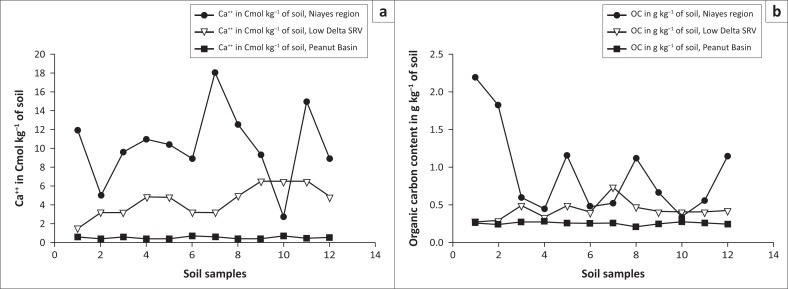
Comparison of calcium contents (a) and organic carbon contents (b) in the Niayes region, the Peanut Basin and the Senegal River Valley.

The monitoring of the dynamics of agricultural lands in the study area highlights a high level of risk to food security occurrence induced by a significant decrease in surface area for agricultural production. For a Sub Saharan developing country with agriculture as a driving economy, any loss of agricultural land constitutes a serious threat to food production (World Bank 2010a). Indeed, in the *Niayes* region, agricultural land has decreased from 2384 ha in 1999 to 1541 ha in 2014. The shrinkage rate has rapidly increased between 2009 and 2014. During this period of time, the losses of agricultural land were evaluated to 556 ha, which represents 65% of the total reduction of the whole land because of a new way of using agricultural lands for habitat areas and/or industrial establishments, which ended up decreasing the horticultural crops production (Figure 5). Therefore, it is predicted that the depletion of agricultural land reserves in the study area in the 2050s will lead to a progressive urbanisation front towards the *Niayes*. Such a phenomenon will, in the end, continue towards other areas such as *Mboro* and *Lompoul*, which are adjacent areas in the same geographical zone as Diamniadio. Consequently, the rural areas in the whole coastal region are thus threatened in the long term (Eriksson & Juhl 2012; World Bank 2003). The probability of the risk of food insecurity is high in the extent to which the soils of the studied area present a high potential of fertility, with neutral soil acidity level (pH 7.0–8.0) and a favourable soil pH range for crop production, compared to relatively high soil acidity levels in the other eco-geographical zones in Senegal (Figure 4). For crop production, the lower the soil acidity, the better the soil conditions for higher crop production. (Diack et al. 2016; Lal 1989). Description of the soil profile (Table 3) presents shallow soil depths, indicating that groundwater in the *Niayes* region is near the soil surface anytime of the year, which is a favourable condition for cropping (Diatta 1996; Sène, Matty & Diatta 2014). Soil colour ranging from light reddish brown to dark greyish brown is an indicator of a good quality soil with hydromorphic features, a relatively high clay content and a stable polyhydric structure (Blavet, Mathe & Leprun 2000; Mauricio & Ildeu 2005; Koné 2007; Koné et al. 2009). The calcium content of the *Niayes* soils is also high, ranging up to 90 % (Figure 6). At the same time, salinity is detected only in trace in the *Niayes* area, while in the lower delta sodium levels can be up to 1.5 Cmol kg^−1^ of soil. In comparison with soils from the Peanut Basin, soils from the *Niayes* area are higher in organic carbon and calcium contents for all samples. Greater organic matter content was noted in the *Niayes* region than in the lower delta of the Senegal River (Feller & Beare 1997). While the organic carbon contents of the soils in the *Niayes* region can be up to 20 g kg^−1^, the variability of such soil properties of the Peanut Basin and the Lower Delta River was between 2 g kg^−1^ and 10 g kg^−1^. This was shown from 80% of the 12 soil samples analysed (Figure 6). When combined, soil pH, clay and fine silt, exchangeable cations and soil organic matter, these physical and chemical properties are considered as pivotal for the sustainability of agricultural production systems (Pieri 1989; Yemefack, Jetten & Rossiter 2006). Indeed, when comparing the soil from the *Niayes* area (reference area) with soils from the lower delta of the Senegal River (Diack & Loum 2014) and those from the Peanut Basin (Aguiar 2009), results show that soils from the *Niayes* region have much better soil quality than the others (Feller 1995; Yao-Kouamé et al. 2008). Higher soil quality, referred to as high soil fertility levels, would result in higher soil productivity (Sanchez 2002) which would ultimately enable an achievement of food security with crops commonly grown in these farmlands on the *Niayes* region. Therefore, a reduction of these farmlands for one reason or another would negatively impact food security achievement. In this regard, using these farmlands, not for crop production (an activity naturally recognised) but to develop urbanisation and to expand industrialisation, would definitely contribute to reducing food insecurity in Senegal, particularly and in the West African region in general.

## Conclusion

Application of optical remote sensing through the processing of satellite images of medium and high spatial resolution (Landsat and SPOT) has made it possible to monitor the changes in agricultural land from 1999 to 2014. This quantification highlights the dynamics of the land use in the *Niayes* area illustrated by a reduction of the surface area, which constitutes a real risk of food insecurity at the national scale. Thus, the loss of agricultural lands of about 100 ha per year in our study area is likely to cause a diminution in the volume of production of fruits and vegetables. The availability of arable lands from the rest of the *Niayes* region is also under threat with population growth and the resulting urbanisation process. The risk of food disaster is a serious concern in that the *Niayes* lands have good soil quality for agricultural purpose compared to soil types from other eco-geographical zones of the country (Peanut Basin and Lower Delta of Senegal River). In addition to the quality of soils, water resources availability with a shallow water table militates in favour of maintaining the use of the *Niayes* area for agricultural purposes. Like the rainfall variability, which is a limiting factor in the performance of agricultural production systems in the Peanut Basin, market gardening and horticulture in the *Niayes* region becomes vulnerable because of the ongoing urbanisation and industrialisation process. Incomes usually generated from the agricultural activities tend to decrease the level of resilience for rural as well as for urban populations. Furthermore, in terms of sustainability, peri-urban agriculture can help mitigate global warming by increasing sequestration of carbon in the soil while industrialisation tends to favour emissions of greenhouse gases in the atmosphere. Therefore, the principles of sustainable development impose the application of public policies pertaining to the conservation of the agricultural land of the *Niayes* region.

## References

[CIT0001] Aguiar L.A, 2009, ‘Impact de la variabilité climatique récente sur les écosystèmes des Niayes du Sénégal entre 1950 ET 2004’, Thèse de Doctorat en Sciences de l’Environnement, Université du Québec, Montréal, pp. 1–185.

[CIT0002] ANACIM, 2015, *Données climatiques et météorologiques de la zone des Niayes sur les cinq dernières années*, Agence Nationale de l’Aviation Civile et de la Météorologie, Dakar, Sénégal.

[CIT0003] Blavet D., Mathe E.E. & Leprun J, 2000, ‘Relation between soils colour and waterlogging duration in a representative hillside of the West African granite-gneissic bedrock’, *Catena* 39(3), 187–210. https://doi.org/10.1016/S0341-8162(99)00087-9

[CIT0004] Benson C. & Clay E.J, 1998, *The impact of drought on sub-Saharan African economies*, Technical paper 401, World Bank, Washington, DC.

[CIT0005] Brooks N, 2003, *Vulnerability, risk, adaptation: A conceptual Framework*, Tyndall Centre for Climate Change Research, University of East Anglia, Working Paper no. XX, 16 p.

[CIT0006] Challinor A., Wheeler T., Garforth C., Craufurd P. & Kassam A, 2007, ‘Assessing the vulnerability of food crop systems in Africa to climate change’, *Climatic Change* 83(3), 381–399. https://doi.org/10.1007/s10584-007-9249-0

[CIT0007] Cooper P.J.M., Dimes J., Rao K.P.C., Shapiro B., Shiferawa B. & Twomlow S, 2008, ‘Coping better with current climatic variability in the rain-fed farming systems of sub-Saharan Africa: An essential first step in adapting to future climate change?’, *Agriculture, Ecosystems and Environment* 126, 24–35. https://doi.org/10.1016/j.agee.2008.01.007

[CIT0008] Cummins D. & Mahul O, 2008, *Catastrophe risk financing in developing countries principles for public intervention overview*, World Bank, Washington, DC.

[CIT0009] Diack M. & Loum M, 2014, ‘Caractérisation par approche géostatistique de la variabilité des propriétés du sol de la ferme agropastorale de l’Université Gaston Berger (UGB) de Saint-Louis, dans le Bas delta du fleuve Sénégal’. *Revue de géographie du laboratoire Leïdi* 12, 1–15.

[CIT0010] Diack M., Loum M., Diom F. & Sow K, 2016, ‘Relationships between soil fertility indicators and toposequence in the Sudano Sahalian watershed of Koutango in the southern peanut basin of Senegal’, *Journal of the Cameroon Academy of Sciences* 13(1–2), 39–47.

[CIT0011] Diatta S, 1996, ‘Les sols gris de bas versant sur granite-gneiss en région centrale de la Cote d’Ivoire: Organisation toposéquentielle et spatiale, fonctionnement hydrologique, conséquence pour la riziculture’, Thèse Université Henri point carré Nancy I, France, 181 p.

[CIT0012] Eriksson J. & Juhl A, 2012, *Guide to risk and vulnerability analyses*, Swedish Civil Contingencies Agency (MSB), Federal Emergency Management Agency (FEMA) (2013) Mitigation Ideas.

[CIT0013] FAO, 2012, *Document stratégique 2012. Plan de réponse face à la crise alimentaire et nutritionnelle au Sahel*, Comite Permanent Inter-Agences (IASC), Dakar, Senegal pp. 1–38.

[CIT0014] Faye E, 2010, ‘Diagnostic partiel de la flore et de la végétation des Niayes et du Bassin arachidier au Sénégal: Application de méthodes floristique, phyto-sociologique, ethnobotanique et cartographique’, Thèse de Doctorat Sciences Agronomiques et Ingénierie Biologique, Université Libre de Bruxelles, pp. 1–253.

[CIT0015] Feller C, 1995, ‘La matière organique du sol: un indicateur de la fertilité. Application aux zones sahélienne et soudanienne’, *Agriculture et Développement* 8, 1–7.

[CIT0016] Feller C. & Beare M.H, 1997, ‘Physical control of soil organic matter dynamics in the tropic’, *Geoderma* 79, 69–116. https://doi.org/10.1016/S0016-7061(97)00039-6

[CIT0017] Gee G.W. & Bauder J.W, 1986, ‘Particle size analysis’, in Klute A. (ed.), *Methods of soil analysis part 2*, 2nd edn, pp. 383–411, Agron. Monog. 9, ASA and SSSA, Madison, WI.

[CIT0018] Giannini A., Biasutti M. & Verstraete M, 2008, ‘A climate model-based review of drought in the Sahel: Desertification, the re-greening and climatic change’, *Global and Planetary Change* 64, 119–128. https://doi.org/10.1016/j.gloplacha.2008.05.004

[CIT0019] Koné B, 2007, ‘La couleur comme indicateur de la fertilité des sols: Utilisation des données pour l’étude de la fertilité potentielle des sols ferrallitiques au-dessus de la latitude 7 degré Nord de la Côte d’Ivoire’, Thèse, Université Cocody, Abidjan, Côte d’Ivoire, 146 p.

[CIT0020] Koné B., Diatta S., Oikeh S., Gbalou Y., Camara M., Dohm D.D. et al., 2009, ‘Estimation de la fertilité potentielle des ferralsols par la couleur: Usage de la couleur en morphopédologie’, *Canadian Journal Soil Science* 89(3), 331–342. https://doi.org/10.4141/CJSS07119

[CIT0021] Lal R, 1989, ‘Land degradation and its impact on food and other resources’, in Pimentel, Hall Carl W. (Eds.), *Food and Natural Ressources* (*1989*), pp. 85–140, Academic Press, New York https://doi.org/10.1016/b978-0-12-556555-4.50009-0

[CIT0022] Leroux M, 1995, ‘La dynamique de la grande sécheresse sahélienne’, *Sécheresse* 70, 223–232.

[CIT0023] Loum M, 2012, ‘Contribution à l’étude de durabilité d’un système de production en milieu sahélien’, Thèse de Doctorat, ENSA de Rennes & UGB Saint-Louis.

[CIT0024] Mauricio P. & Ildeu A, 2005, ‘Color attributes and mineralogical characteristics, evaluated by radiometry of highly weathered tropical soils’, *Soil Science Society of America Journal* 69, 1162–1172. https://doi.org/10.2136/sssaj2003.0312

[CIT0025] Mclean E.O, 1982, *Soil pH and lime requirement*, Agron. 9, pp. 199–224, SSSA Madison, WI.

[CIT0026] Ndao M, 2012, ‘Dynamiques et gestions environnementales des zones humides au Sénégal: étude de l’occupation du sol par télédétection des Niayes’, Université de Toulouse Mirail et UGB Saint-Louis, pp. 1–370.

[CIT0027] Nelson D.W. & Sommers L.E, 1982, ‘Total carbon, organic carbon and organic matter’, in Page A.L. (ed.), *Methods of soil analysis. Part 2: Chemical and microbiological properties*, pp. 259–579, Agron. Journal. 9, Madison, WI.

[CIT0028] Pieri C, 1989, *Fertilité des terres de savanes: bilan de trente ans de recherche et de développement agricole au sud du Sahara*, pp. 1–444, Ministère de la coopération française, CIRAD-IRAT, Montpellier, France.

[CIT0029] Poncet Y, 1986, *Images spatiales et Paysages sahéliennes, une étude des milieux naturels par télédétection*, Azawagh, République du Niger, pp. 1–235, ORSTOM, Montpellir, France.

[CIT0030] Sanchez P.A, 2002, ‘Soil fertility and hunger in Africa’, *Science* 295(5562), 2019–2020. https://doi.org/10.1126/science.1065256 11896257 10.1126/science.1065256

[CIT0031] Seck M., Abou M.M., Wade S. & Thomas J.P, 2005, *L’étude de cas des systèmes de production agricoles de Sébikotane (Sénégal)*, pp. 1–33, ENDA Tiers Monde, Dakar Senegal.

[CIT0032] Sène J.H.B., Matty F. & Diatta M, 2014, ‘Caractérisation des sols de la vallée rizicole de Tamra, dans l’ile de Mar, Centre-Ouest du Sénégal’, *International Journal of Biological and Chemical Sciences* 8(2), 794–810. https://doi.org/10.4314/ijbcs.v8i2.35

[CIT0033] Toupet C, 1995, ‘La crise sahélienne’, *Revue de géographie de Lyon* 70(3–4), 181–186.

[CIT0034] Veyret Y. & Reghezza M, 2005, ‘Aléas et risques dans l’analyse géographique’, *Annales des Mines* 61–69.

[CIT0035] Wade I., Benz H.D. & Egg J, 2004, ‘Information et régulation des filières maraîchères au Sénégal’, *Cahiers Agricultures* 13(1), 148–157.

[CIT0036] World Bank, 2003, ‘Building safer cities’, in Kreimer A., Arnold M. & Carlin A. (eds.), pp. 3–298, *The future of disaster risk*, World Bank, Washington, DC.

[CIT0037] World Bank, 2010a, *Report on the status of disaster risk reduction in sub-Saharan Africa*, pp. 1–44, Global Facility Reduction and Recovery, World Bank, Washington, DC The African Centre of Meteorological Applications for Development. (ACMAD).

[CIT0038] World Bank, 2010b, *Disaster risk management in Senegal*, World Bank, Washington, DC.

[CIT0039] Yao-Kouamé A., Yao G.F., Alui K.A., N’guessan A.K., Tiemoko T.P. & Yao K.K, 2008, ‘Etude morphopédologique du bassin versant du mont Blanguand dans le massif du Yaouré en région centre de la Côte d’Ivoire’, *Afrique Science* 4(3), 426–451.

[CIT0040] Yemefack M., Jetten V.G. & Rossiter D.G, 2006, ‘Developing a minimum data set for characterizing soil dynamics in shifting cultivation systems’, *Soil and Tillage Research* 86, 84–98. https://doi.org/10.1016/j.still.2005.02.017

